# Activity of lumacaftor is not conserved in zebrafish Cftr bearing the major cystic fibrosis‐causing mutation

**DOI:** 10.1096/fba.2019-00039

**Published:** 2019-09-18

**Authors:** Onofrio Laselva, Steven Erwood, Kai Du, Zhenya Ivakine, Christine E. Bear

**Affiliations:** ^1^ Programme in Molecular Medicine Hospital for Sick Children Toronto Canada; ^2^ Department of Physiology University of Toronto Toronto Canada; ^3^ Programme in Genetics and Genome Biology Hospital for Sick Children Toronto Canada; ^4^ Department of Biochemistry University of Toronto Toronto Canada

**Keywords:** ABC transporters, CFTR, CFTR modulators, cystic fibrosis, F508del‐CFTR, zebrafish

## Abstract

F508del‐cystic fibrosis transmembrane conductance regulator (CFTR) is the major mutant responsible for cystic fibrosis (CF). ORKAMBI^®^, approved for patients bearing this mutant, contains lumacaftor (VX‐809) that partially corrects F508del‐CFTR's processing defect and ivacaftor (VX‐770) that potentiates its defective channel activity. Unfortunately, the clinical efficacy of ORKAMBI^®^ is modest, highlighting the need to understand how the small molecules work so that superior compounds can be developed. Because, human CFTR (hCFTR) and zebrafish Cftr (zCftr) are structurally conserved as determined in recent cryo‐EM structural models, we hypothesized that the consequences of the major mutation and small molecule modulators would be similar for the two species of protein. As expected, like the F508del mutation in hCFTR, the homologous mutation in zCftr (F507del) is misprocessed, yet not as severely as the human mutant and this defect was restored by low‐temperature (27°C) culture conditions. After rescue to the cell surface, F507del‐zCftr exhibited regulated channel activity that was potentiated by ivacaftor. Surprisingly, lumacaftor failed to rescue misprocessing of the F507del‐zCftr at either 37 or 27°C suggesting that future comparative studies with F508del‐hCFTR would provide insight into its structure: function relationships. Interestingly, the robust rescue of F508del‐zCftr at 27°C and availability of methods for in vivo screening in zebrafish present the opportunity to define the cellular pathways underlying rescue.

**Abbreviations**
cAMP: cyclic adenosine monophosphate
CF: cystic fibrosis
CFTR: cystic fibrosis transmembrane conductance regulator
CHX: cycloheximide
FLIPR: fluorometric imaging plate reader
FSK: forskolin
GFP: green fluorescent protein
HEK293: human embryonic kidney 293
MSD: membrane‐spanning domain
NBD: nucleotide‐binding domain
RIPA: radioimmunoprecipitation assay
WT: wild‐type

## 1 INTRODUCTION

Cystic fibrosis (CF), the most prevalent genetic disease in Caucasians,^1^, ^2^ is caused by mutations in the *CFTR* gene and the loss of function of the cystic fibrosis transmembrane conductance regulator (CFTR) protein.^3^ CFTR is member of the ABC superfamily of transporters and is a unique family member as it functions as an adenosine triphosphate (ATP)‐ and protein kinase A (PKA)‐activated anion channel.^3^ Normally, the CFTR channel mediates chloride flux across apical membranes of polarized epithelial cells in certain tissues including the lung, liver, intestine, pancreas, and sweat glands.^3^, ^4^, ^5^ In the airway epithelium, the lack of CFTR channel function on the cell surface causes impaired mucociliary clearance, recurrent bacterial infections, and progressive deterioration of lung function.^6^, ^7^

The CF field recognizes the importance of understanding the molecular mechanisms governing CFTR biosynthesis and function in order to define its role in cellular biology and identify targeted therapeutic strategies for CFTR mutants. The first topological models have been largely validated.^8^ The CFTR protein consists of two membrane‐spanning domains (MSD1, MSD2) with four intracellular loops (ICL1‐4), two nucleotide‐binding domains (NBD1, NBD2), and a regulatory (R) domain that harbors most of the PKA sites responsible for the phosphorylation‐dependent regulation of CFTR channel opening.^8^ Initially, the tertiary structure of CFTR and assembly of the above domains were modeled using crystal structures of related ABC proteins.^9^, ^10^, ^11^ Recently, the structure of zebrafish (zCftr) and human (hCFTR) in the dephosphorylated “closed” channel state and in the phosphorylated “open” channel state were solved using cryo‐EM.^12^, ^13^, ^14^, ^15^ The availability of structures from both species, sharing 55% sequence homology, provides the opportunity for detailed structure: function analyses. Recent patch‐clamp studies showed both species of CFTR exhibited ATP‐ and phosphorylation‐dependent anion channel activity, albeit with variation in gating kinetics.^16^ These electrophysiological studies support previous in vivo, phenotypic studies of Cftr in zebrafish showing that it normally functions as an anion channel and drives pancreatic fluid transport.^17^, ^18^

While it has been shown that most of the residues that are mutated and cause CF disease are conserved between zCftr and hCFTR,^12^, ^14^ it is not known if the consequences of these mutations are identical between the two species. It is particularly important to determine if the zebrafish protein bearing the major mutation causing CF recapitulates the primary defects and pharmacological responses observed in the mutant human protein (F508del‐hCFTR) if it is to be useful in modeling these activities.

The most common CF‐causing mutation results in deletion of phenylalanine at residue 508 (F508del‐hCFTR) in NBD1 of hCFTR. This mutation causes defective folding of NBD1 and alters its assembly with the rest of the protein.^19^, ^20^, ^21^, ^22^ The misassembled mutant protein is misprocessed and retained in the endoplasmic reticulum.^23^, ^24^, ^25^ However, it has been shown that culturing cells at a low temperature (27°C) can rescue the trafficking defect of F508del‐hCFTR and its expression at the cell surface, although rescued channels still exhibit temperature‐dependent defects in gating and stability.^26^, ^27^, ^28^ It remains to be determined if deletion of phenylalanine at the same position on NBD1 of zCftr will induce a similar molecular phenotype and enable the modeling of therapeutic strategies for the major mutation.

ORKAMBI^®^ is approved for the treatment of individuals who are homozygous for the F508del mutation. It is a combination of lumacaftor (VX‐809), a corrector compound that partially rescues the biosynthetic defect of F508del‐hCFTR, and ivacaftor (VX‐770), a small molecules that potentiates the channel activity of the mutant protein once rescued to the cell surface.^29^, ^30^ While ORKAMBI^®^ has been shown to improve FEV1 in CF patients homozygous for F508del mutation,^31^ the response size is modest and variable, prompting the need to understand its mechanism of action to aid in future drug discovery.

Biochemical and biophysical studies suggest that VX‐809 and VX‐770 interact directly with the hCFTR protein.^32^, ^33^, ^34^, ^35^, ^36^, ^37^, ^38^, ^39^, ^40^ Molecular docking studies and mutagenesis studies have implicated potential binding sites for VX‐809, yet uncertainty remains, as no direct binding assays have yet been developed. On the other hand, recent cryo‐electron microscopy studies have modeled the binding site for VX‐770 at a lipid protein interface docking into a groove formed by transmembrane (TM) helices 4, 5, and 8.^41^

In this paper, we show that introduction of the major CF‐causing mutation into zCftr recapitulates the consequences observed in the hCFTR protein but fails to model the pharmacological response to VX‐809.

## 2 MATERIAL AND METHODS

### 2.1 Cell culture and transfection

Human and Zebrafish CFTR used in this study were transiently expressed in human embryonic kidney 293 (HEK293) GripTite cells (HEK293) from Dr Daniela Rotin, Hospital for Sick Children, Toronto, Ontario, Canada. Cells were maintained in DMEM (Wisent) supplemented with non‐essential amino acids (Life Technologies) and 10% fetal bovine serum (FBS; Wisent) at 37 or 27°C with 5% CO_2_ (HEPA incubator, Thermo Electron Corporation) and processed as previously described.^42^, ^43^ Transient transfections were performed using PolyFect Transfection Reagent (Qiagen) according to the manufacturer's protocol, as previously described.^43^

### 2.2 Plasmids, antibodies, and reagents

The human green fluorescent protein (GFP)‐tagged CFTR cDNA constructs were generated using InFusion cloning (ClonTech). In brief, a GFP tag with a 20 amino acid linker was cloned onto the C‐terminus of two constructs containing either wild‐type (WT)‐CFTR or F508del‐CFTR. The GFP tag with its accompanying linker was amplified with primers that incorporated 15 bp of flanking overlap corresponding to the destination vector from a construct containing the cDNA of GFP fused zCftr, which has been previously described.^17^ This was then cloned via InFusion cloning into WT CFTR and CFTR F508del vectors that were linearized by PCR.

Zebrafish WT‐Cftr C‐terminus GFP tagged was kindly provided by M. Bagnat (Duke University School of Medicine).^17^ Mutated CFTR was generated using the KAPA HiFi HotStart PCR Kit (KAPA BIOSYSTEMS) according to the manufacturer's standard PCR protocol with high‐quality (>300 ng/µL, 260/280 nm ratio of 1.8) plasmid DNA, containing zebrafish WT‐Cftr‐GFP (in pcDNA3.1) as the template, as previously described.^43^, ^44^

The primary antibodies used in this study were rabbit Ab Anti‐GFP (Abcam) for F508del‐hCFTR‐GFP and zCftr constructs and mouse Ab Anti‐CFTR 596 (University of North Carolina Chapel Hill) for hCFTR constructs. Calnexin was used as a protein loading control and detected with a calnexin‐specific rabbit pAb (Sigma‐Aldrich).

The small molecule modulators of CFTR used in this study were: VX‐770 and VX‐809 (Selleck Chemicals); the CFTR inhibitor, CFTR_inh_‐172 (Cystic Fibrosis Foundation Therapeutics).

### 2.3 Immunoblotting

Human embryonic kidney 293 cells were transiently transfected with the plasmids as described above. After 18 hours transfection, cells transfected with WT‐hCFTR‐GFP, F508del‐hCFTR, F508del‐hCFTR‐GFP, or F507del‐zCftr‐GFP were treated with 3 μM VX‐809 or DMSO for 24 hours at 27 or 37°C. Then, cells were lysed in modified radioimmunoprecipitation assay (RIPA) buffer (50 mmol/L Tris‐HCl, 150 mmol/L NaCl, 1 mmol/L EDTA, pH 7.4, 0.2% Sodium dodecyl sulfate (SDS), and 0.1% Triton X‐100) containing a protease inhibitor cocktail (Roche) for 10 minutes. Soluble fractions were analyzed by SDS‐PAGE on 6% Tris‐Glycine gels (Life Technologies). After electrophoresis, proteins were transferred to nitrocellulose membranes (Bio‐Rad) and incubated in 5% milk. hCFTR was detected with hCFTR‐specific murine mAb 596 (1:5000) or rabbit Ab Anti‐GFP (1:5000). The blots were developed with ECL (Amersham) on the Li‐Cor Odyssey Fc (LI‐COR Biosciences) in a linear range of exposure.^44^, ^45^, ^46^, ^47^

To evaluate protein glycosylation status, HEK293 cells were transiently transfected with WT‐zCftr‐GFP or F507del‐zCftr‐GFP. The cells were grown at 37°C for 24 hours and subsequently lysed in modified RIPA buffer as described above. Lysates were treated with either 500 U of endoglycosidase H or 500 U of peptide‐N‐glycosidase F (both from New England Biolabs) according to the manufacturer's protocol. Immunoblots were obtained using rabbit Ab Anti‐GFP as described above.

### 2.4 Functional measurements

Cystic fibrosis transmembrane conductance regulator activity was measured using a membrane depolarization assay (fluorometric imaging plate reader [FLIPR]) as previously described.^43^, ^44^, ^48^ Briefly, HEK293 cells were grown at 37°C in 96‐well plates (Costar). The cells were transfected with F508del‐hCFTR‐GFP, WT‐zCftr‐GFP, F507del‐zCftr‐GFP. After 18 hours, the cells expressing F508del‐hCFTR‐GFP or F507del‐zCftr‐GFP were treated with 3 μM VX‐809 or DMSO for 24 hours at 37 or 27°C. The cells were then washed with PBS and the blue membrane potential dye (dissolved in chloride‐free buffer as described,^43^, ^44^, ^49^) which can detect changes in TM potential, was added to the cells for 40 minutes at 37 or 27°C. Changes in fluorescence were detected using a fluorescence microplate reader (SpectraMax i3; Molecular Devices) at 37 or 27°C.

After 5 minutes baseline, CFTR was stimulated using the cyclic adenosine monophosphate (cAMP)‐agonist forskolin (FSK) (1 or 10 μM; Sigma) or FSK in combination with VX‐770 (1 μmol/L, Selleckchem). The CFTR inhibitor CFTR_inh_‐172 (10 μM, Cystic Fibrosis Foundation Therapeutics) was added to terminate the measurement. CFTR activation was measured as the difference in the maximum rate of change of the FLIPR signal after addition of agonist ± potentiator relative to the baseline rate of FLIPR signal change.

### 2.5 Measurement of CFTR stability

Human embryonic kidney 293 cells were transiently transfected with F508del‐hCFTR or F507del‐zCFTR‐GFP as described above. After 24 hours incubation at 27°C, protein synthesis was stopped by addition of the medium containing 0.5 mg/mL cycloheximide (CHX) and immediately incubated at 37°C for up to 8 hours. Whole cell extracts obtained at various time periods (0‐8 hours) were subjected to western blot analysis.^44^

### 2.6 Statistical analysis

Data are presented as mean ± SEM unless otherwise noted. GraphPad Prism 7.0 software (San Diego, CA) was used for all statistical analysis. Student's t tests, one‐way/two‐way ANOVA were conducted as appropriate, and *P*‐values < 0.05 were considered significant. Data with multiple comparisons were assessed using Tukey's multiple‐comparison test with *α* = 0.05. Each experiment conducted on independently plated cells, was defined as a separate biological study.

## 3 RESULTS

### 3.1 Expression and functional characterization of zebrafish WT‐zCftr

**Figure 1:**
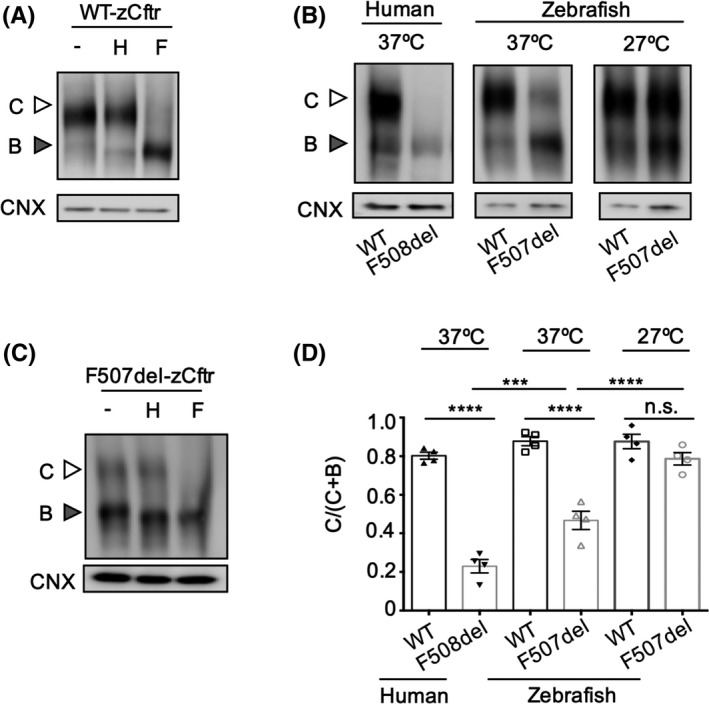
The processing defect of F507‐zCftr is fully rescued by low temperature correction at 27°C. A, HEK293 cells were transiently transfected with WT‐zCftr‐GFP. To evaluate glycosylation status, immunoblots show WT‐zCftr‐GFP control (−) and its sensitivity to endoglycosidase H (H) and peptide‐N‐glycosidase F (F). White arrowhead, complex glycosylated (Band C); gray arrowhead, core glycosylated (Band B). The zCftr‐GFP was detected with an antibody against GFP. B, HEK293 cells were transiently transfected with WT‐hCFTR‐GFP, F508del‐hCFTR‐GFP, WT‐zCftr‐GFP, or F507del‐zCftr‐GFP at 37°C (left) or 27°C (right) (n = 4). The zebrafish mutant recapitulates the primary defect in processing observed in the human mutant at 37°C. C, Immunoblots show F507del‐zCftr‐GFP protein exhibit partial processing to mature form, Band C at 37ºC, as evident in its resistance to endoglycosidase H (H) and sensitivity to peptide‐N‐glycosidase F (F). D, Bar graphs show the mean (±SEM) of the ratio (Band C)/(Band (C + B)) of the WT and F508del‐hCFTR‐GFP proteins plus WT and F507del‐zCftr‐GFP protein at 37 and 27°C (n = 4) (***P < .001; ****P < .0001). Here and throughout the rest of the text, all of the Wt and mutant zCftr constructs bear a carboxy terminus GFP tag. Calnexin (CNX) was used as a loading control. GFP, green fluorescent protein; hCFTR, human CFTR; HEK293, human embryonic kidney 293; WT, wild‐type

In order to study the expression and function of zCftr in HEK‐293 cells, we obtained the cDNA‐coding zCftr (WT) with a C‐terminal GFP tag from M. Bagnat (Duke University). As none of the commercially available antibodies targeting hCFTR recognized the zCftr protein, the GFP tag enabled the study of the zCftr protein by immunoblotting using an anti‐GFP antibody. The GFP‐tagged zCftr protein migrated as two bands corresponding to the core (Band B) and complex‐glycosylated (Band C) form of the protein (Figure 1A).^50^ As expected, the C band of the zebrafish protein was insensitive to endoglycosidase H, yet sensitive to peptide‐N‐glycosidase F, indicative of complex glycosylation (Figure 1A). We confirmed that the zebrafish protein exhibited FSK‐dependent chloride channel activity, similar to that of the hCFTR protein, both bearing a carboxy‐terminal GFP tag (Figure S1A,B). Moreover, we confirmed that the carboxy‐terminal GFP tag did not alter the FSK‐dependent channel activation of hCFTR as studied in HEK293 cells using a fluorescence membrane potential dye assay (FLIPR) (Figure S1C).

Deletion of F508 in hCFTR leads to misfolding, misassembly, and misprocessing.^5^, ^51^ As shown in Figure 1B, F508Del‐hCFTR‐GFP exhibits this protein misfolding defect. Structural alignment with zCftr reveals that the corresponding residue in zCftr is F507. We found that F507del‐zCftr is also misprocessed relative to WT zebrafish protein. Interestingly, unlike the mutant hCFTR, the mutant zCftr exhibits partial processing to the mature Band C form of the protein when expressed in HEK293 cells at 37°C (Figure 1B,D). The identity of Band C of F507del‐zCFTR was confirmed in glycosidase studies, showing its resistance to EndoH and sensitivity to peptide‐N‐glycosidase F (Figure 1C).

It has been shown that culturing cells at low temperature (27°C) can rescue the functional expression of F508del‐hCFTR at the cell surface, although the protein still displays an altered rate of channel activation with FSK and reduced stability at the cell surface.^26^, ^27^, ^28^ We investigated the effect of low temperature correction on F507del‐zCftr. Interestingly, we found that the processing defect of F507del‐zCftr was fully rescued by low temperature correction at 27°C for 24 hours (Figure 1B‐D) with the complex‐glycosylated form constituting the dominant form of the protein at this temperature.

**Figure 2:**
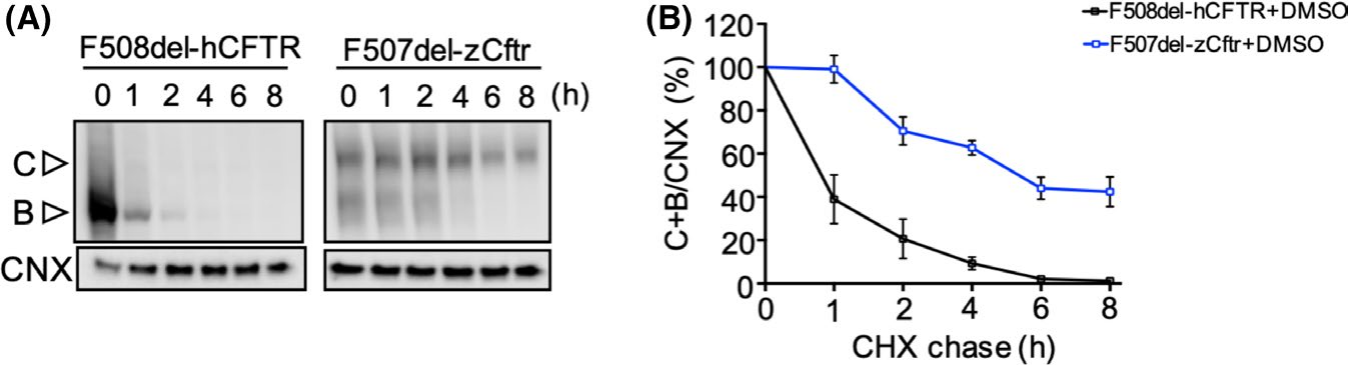
At low temperature, F507del‐zCftr generates a stable mature complex‐glycosylated protein. A, F508del‐hCFTR or F507del‐zCftr‐GFP was expressed in HEK293 cells. After 24 h incubation at 27°C, protein synthesis was inhibited by addition of CHX (0.5 mg/mL) and then the cells were shifted to 37°C to monitor thermal stability. Cells were collected after the indicated times for western blot analysis of whole cell extracts. B, The total amount of F508del‐hCFTR or F507del‐zCftr protein at each time point was quantitated and expressed relative to that at time 0 and calnexin loading (n = 4). CHX, cycloheximide; GFP, green fluorescent protein; hCFTR, human CFTR; HEK293, human embryonic kidney 293

Then, we compared the stability of the temperature rescued, F508del‐hCFTR, and F507del‐zCftr proteins by immunoblotting and CHX chase after low temperature rescue for 24 hours. In Figure 2A,B, we show that the turnover of the total F508del‐hCFTR was rapid, as expected, with more than half the protein degraded within 1 hour. On the other hand, the F507del‐zCftr was much more stable, with 50% remaining after 8 hours.

### 3.2 The F507del‐zCftr channel activity is impaired relative to WT‐zCftr and is potentiated by VX‐770

**Figure 3:**
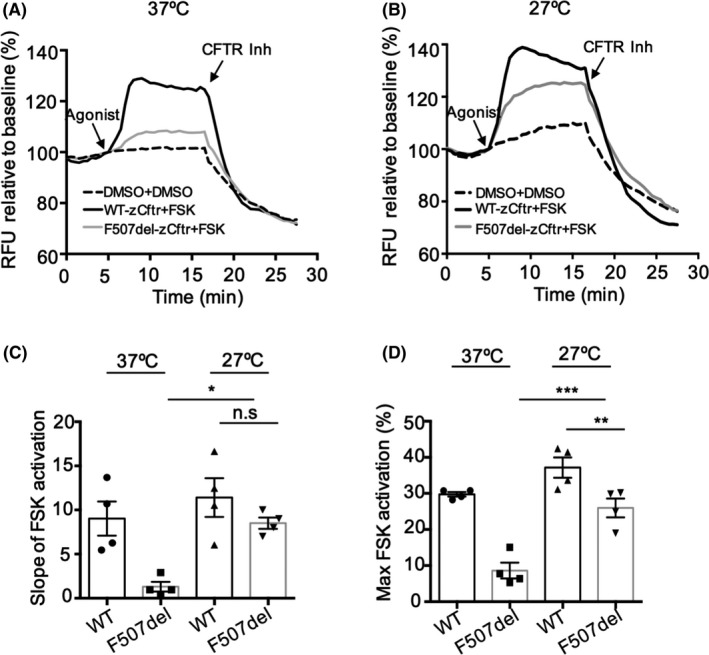
Activation of F507del‐zCftr exhibits full rescue after low temperature rescue. Functional analysis using the fluorometric imaging plate reader membrane depolarization assay of WT‐or F507del‐zCftr‐GFP in the presence of 0.1% DMSO or 10 μM of FSK in HEK293 cells at 37°C (A) or 27°C (B). After 10 min activation by FSK, CFTR Inhibitor (CFTR_inh_‐172, 10 μM) was added to deactivate CFTR. (C) Bar graph shows the mean (±SEM) of the slope of FSK activation at 37°C and 27°C (n = 4). (D) Bar graphs show the mean (±SEM) of maximal activation of CFTR after stimulation by FSK at 37 and 27°C (n = 4). (*P < .05, **P < .01, ***P < .001). CFTR, cystic fibrosis transmembrane conductance regulator; FSK, forskolin; GFP, green fluorescent protein; HEK293, human embryonic kidney 293; WT, wild‐type

We investigated the channel activity of F507del‐zCftr using a membrane potential dye assay (FLIPR) in HEK293 cells. We demonstrated that the cAMP‐dependent conduction conferred by F507del‐zCftr in the macroscopic FLIPR assay (at 37°C) was severely impaired, relative to WT‐zCftr (Figure 3A‐D).^23^ At low‐temperature preincubation, the rate of activation and peak levels of F507del‐zCftr channel function are increased to approximately 60%‐70% of those observed for the WT‐zCftr protein (Figure 3B‐D). These findings suggest that low temperature and the resulting changes in proteostatic regulatory interactions with the mutant zCftr are very effective in correcting the consequences of F507del on the assembly of the zCftr.

**Figure 4:**
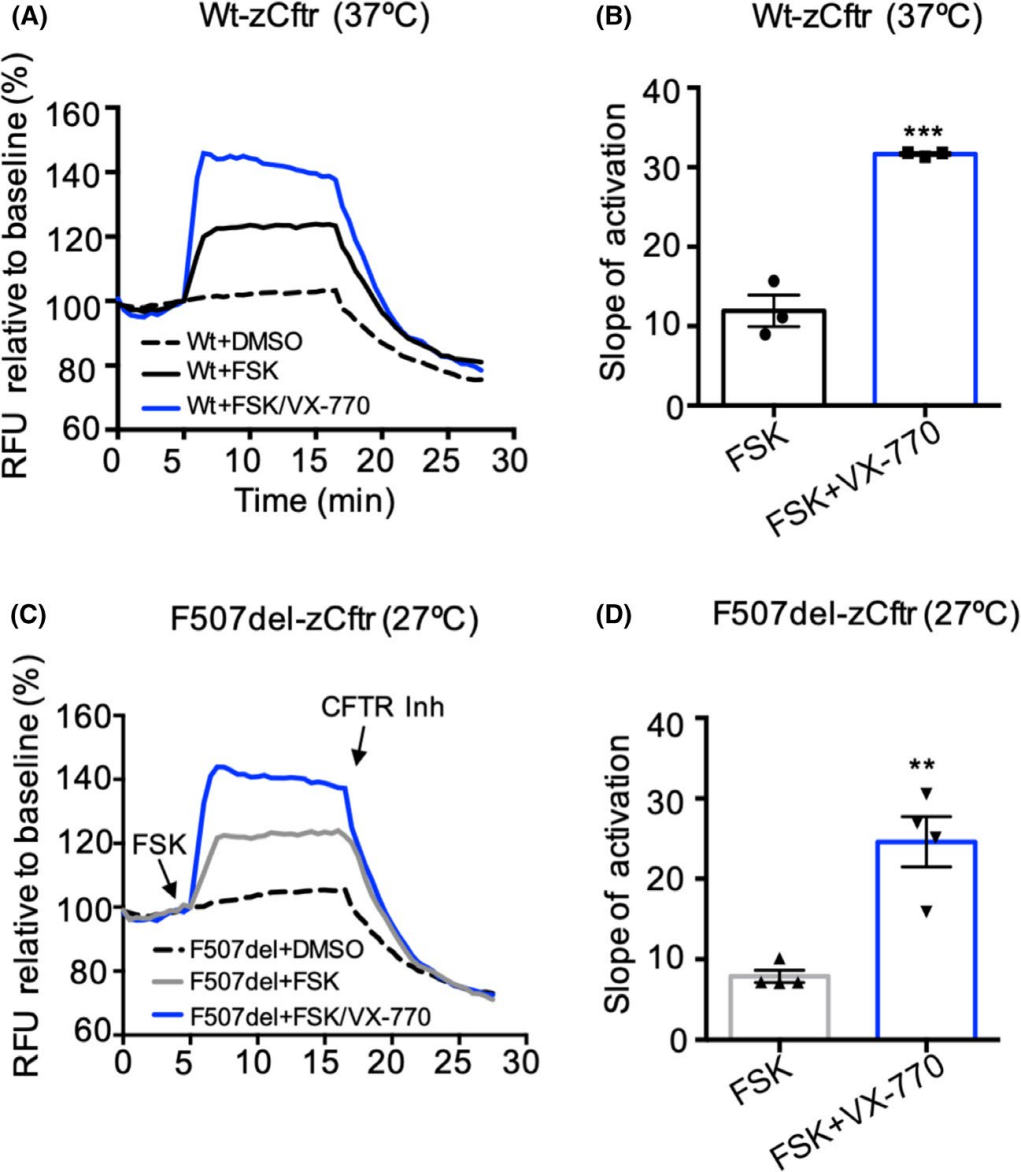
WT‐zCftr and F507del‐zCftr channel activity can be potentiated by VX‐770. A, Functional analysis of WT‐zCftr‐GFP in HEK293 cells using FLIPR assay by 0.1% DMSO or 1 μM FSK in the presence or absence of 1 μM VX‐770, at 37°C. After 10 min activation by FSK, CFTR Inhibitor (CFTR_inh_‐172, 10 μM) was added to deactivate CFTR. B, Bar graph shows the mean (±SEM) of the slope of FSK activation at 37°C (n = 4). C, Representative traces of F507del‐zCftr‐GFP function following 24 h preincubation at 27°C and acute activation with 1 μM FSK ± 1 μM VX‐770 at 27°C. D, Bar graph shows the mean (±SEM) of the slope of FSK activation at 27°C (n = 4). (**P < .01, ***P < .001). CFTR, cystic fibrosis transmembrane conductance regulator; FLIPR, fluorometric imaging plate reader; FSK, forskolin; GFP, green fluorescent protein; HEK293, human embryonic kidney 293; WT, wild‐type

KALYDECO^™^, also known as ivacaftor (VX‐770), is a small molecule “potentiator” that promotes the open state of the hCFTR channel.^29^ Here, we investigated the effect of VX‐770 on the channel activity of WT‐zCftr. As shown in Figures 1, 4A,B, 1 μM VX‐770 potentiated WT‐zCftr‐dependent chloride efflux. VX‐770 potentiated WT‐zCftr‐dependent chloride efflux, with the same affinity as measured for WT‐hCFTR (Figure S2A). Moreover, VX‐770 potentiates F507del‐zCftr after its rescue to the cell surface at 27°C (Figure 4C,D). Therefore, the molecular determinants of VX‐770 potentiation are conserved in both species of CFTR as expected on the basis of the primary sequence conservation at the binding site identified in recent cryo‐electron studies.^41^

### 3.3 Lack of expression and functional F507del‐zCFTR rescue at 37°C by VX‐809

Previous studies showed that VX‐809 partially rescues the misprocessing of F508del‐hCFTR, via stabilization of MSD1 and rescue of aberrant interactions between the MSDs and F508del‐NBD1. As a result of these conformational changes, forward trafficking of the full length mutant through the biosynthetic pathway is promoted where it can be activated by FSK and potentiated by VX‐770 at the plasma membrane.^30^, ^35^, ^36^, ^40^, ^44^ In this study, we investigated the effect of VX‐809 on F507del‐zCftr processing at 37°C.

**Figure 5:**
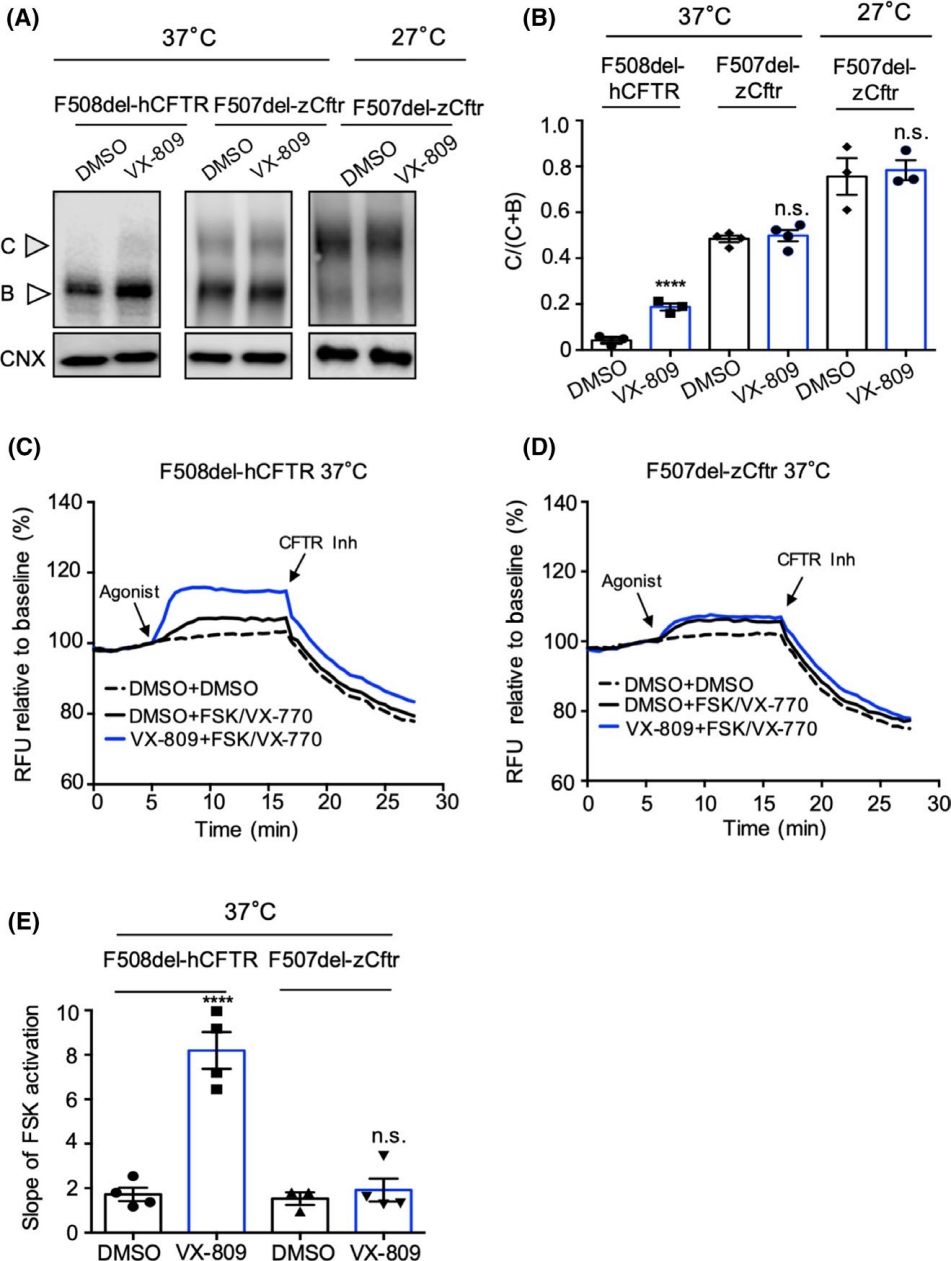
At 37°C, zebrafish Cftr (F507del) exhibits negligible rescue of the processing defect with VX‐809. A, HEK cells were transiently transfected with F508del‐hCFTR‐GFP (left) and F507del‐zCftr‐GFP (right two panels), in the presence or absence of 3 μM VX‐809 at 37 or 27°C. Band C, fully processed, mature complex‐glycosylated CFTR; Band B, immature core‐glycosylated CFTR are indicated. Unlike the human mutant, there is no positive effect of VX‐809 on processing of the zebrafish mutant. B, Bar graphs show the quantification of immunoblots like those shown in A) for four biological repeats. The bars show mean (±SEM) of the ratio C/(C + B) for the human and zebrafish mutants. C, Representative traces (membrane depolarization assay) for F508del‐hCFTR‐GFP channel activation and potentiation, with or without VX‐809 pretreatment. In these experiments, CFTR‐mediated depolarization was inhibited with the addition of CFTR_inh_‐172 (10 µM). D, Unlike the human mutant, there is no effect of VX‐809 on activated and potentiated F507del‐zCftr‐GFP channel function following chronic treatment with 3 μM VX‐809. E, Bar graph shows the mean (±SEM) of the initial slope after potentiation at 37°C (n = 4). Calnexin (CNX) was used as a loading control. (**P < .01). GFP, green fluorescent protein; hCFTR, human CFTR; cystic fibrosis transmembrane conductance regulator; HEK, human embryonic kidney

Here, we showed that preincubation with VX‐809 for 24 hours was able to enhance the processing of F508del‐hCFTR‐GFP but was ineffective in F507del‐zCftr‐GFP at either 37 or 27°C (Figure 5A‐E). These findings suggest that the zebrafish mutant is lacking the VX‐809 binding site or its defective in its mechanism of action. Interestingly, despite higher constitutive levels of mature F507del‐zCftr‐GFP protein than the F508del‐hCFTR‐GFP at 37°C (Figure 5B), the zebrafish mutant failed to exhibit potentiated channel activity (Figure 5C,E). These results suggest that even the complex‐glycosylated form of the mutant channel lacks normal modulation at 37ºC, a temperature that exceeds the normal range of bodily temperatures in zebrafish (ranging from 21 to 32°C^52^).

## 4 DISCUSSION

These studies show that certain primary defects caused by deletion of F507 (the residue in zCftr that aligns with F508 in hCFTR), recapitulate those previously described for deletion of F508 in the hCFTR protein.^53^, ^54^ Specifically, the mutant zCftr protein is misprocessed at 37°C and shows reduced functional expression as a chloride channel. Reduction of the cell culture temperature is more effective in rescuing these primary defects for the zebrafish mutant than for the human mutant. On the other hand, the F507del‐zCftr is not responsive to the small molecule corrector, lumacaftor, that partially rescues F508del‐CFTR and together with the potentiator, VX‐770, is currently in use in CF patients. These findings show that, despite the structural conservation of these two proteins with 55% sequence identity, the consequences of the single‐site deletion, temperature‐dependent proteostatic mechanisms, and consequences of lumacaftor binding are different between the species. Therefore, these findings will guide future studies of the temperature‐dependent protein interactions that modulate mutant CFTR protein folding and processing as well as the structural basis for lumacaftor‐mediated correction.

As previously mentioned, the F507del mutation induces misprocessing in the zebrafish protein.^5^, ^51^ However, the misprocessing defect in delta F507del‐zCftr is not as severe as the defect induced by delta F508 in hCFTR with the C/C + B ratios for the zebrafish and human proteins being 43% and 16% of WT, respectively (see Figure 1A). Previous studies^53^, ^54^, ^55^, ^56^ have highlighted differences in CFTR function, pharmacology, and the consequences of the F508del mutation across species. Some amelioration of the misprocessing defect, as shown in this study, has also been seen for other species including mouse, ferret, and pig.^54^ This has been attributed to the presence of second‐site amino acid substitutions, so‐called revertant mutations. In fact, zCftr possesses two of the revertant mutants that are widely cited in the CF literature (A534P and I539T) and that have previously been used to improve the stability of the hCFTR protein for structural studies.^57^, ^58^, ^59^

Previous studies have demonstrated that the revertant I539T mutation partially improved F508del‐hCFTR processing, thermostability, and channel gating suggesting that correction of NBD1 rescues the misfolding of F508del‐hCFTR.^60^, ^61^ In pilot experiments, we found that there is an amino acid substitution in NBD1 at position 539 of zCFTR that may contribute to partial processing to the mature Band C like the other species^53^, ^54^, ^58^ (Figure S4A). Here, we confirmed that substitution of threonine at this position in the mutant zebrafish protein with isoleucine (as in the human sequence) abrogated residual processing (Figure S4A‐C). We will test our prediction that the substitution impairs channel gating in the mutant zebrafish protein in future work.

Interestingly, despite the greater abundance of mature mutant zCftr protein relative to mature mutant hCFTR at 37°C, F507del‐zCftr fails to exhibit greater FSK‐activated channel activity. Recently, it has been shown that the WT‐zCftr channel has a low open probability and reduced unitary conductance relative to the hCFTR channel.^16^ Although the single channel properties of the mutant zCftr are unknown, it is possible that its open probability and unitary conductance are further reduced from the WT‐zCftr rendering channel activation of the mutant very difficult to detect.

Interestingly, VX‐809 was ineffective in enhancing the processing and functional expression of delta F507del‐zCftr relative to the vehicle control (DMSO) (Figure 5). With approximately 55% sequence identity between the zebrafish and human proteins, this finding may reflect the lack of conservation of the VX‐809 binding site and/or non‐conservation of the conformational changes induced by VX‐809 that are important for its activity. On the other hand, F508del‐mCftr, with 78% sequence identity with the human protein, does show rescue of its processing defect with VX‐809, arguing that comparative studies of the zebrafish, mouse, and hCFTR proteins may inform the mechanism of action of this corrector.^62^

Although, the binding site for VX‐809 has not been fully defined, regions of a putative pocket were implicated in biochemical studies (ie, MSD1,^35^, ^40^) or in silico studies (NBD1 and the coupling helices extending from MSD1 and MSD2,^59^, ^63^, ^64^, ^65^). Although some of the residues highlighted in previous studies are conserved between the human and zebrafish proteins (ie, R170, F374, L375, E403, and R1070), others are not (i.e, E402, V510, E474, G1069). Interestingly, the specific residues in the hCFTR that were implicated in the VX‐809 binding site by the Cyr group, namely 374 and 375 on the cytosolic extension of TM segment 6, are conserved in the zebrafish protein.^35^ On the other hand, sites that are modeled at the interface between NBD1 and the coupling helix of ICL4 and proposed to be disrupted in the mutant and repaired by VX‐809 are not completely conserved. For example, valine at position 510 on NBD1 in the human protein is substituted with leucine. The apposing residue on ICL4 in the human protein, that is, glycine at position 1069 is substituted with glutamate. On the other hand, the arginine residue at position 1070, proposed to partially mediate folding of CFTR, is conserved in the zebrafish protein.^63^, ^65^ Therefore, it remains unclear if the lack of VX‐809 effect on F507del‐Cftr reflects defective binding and/or altered conformational response to its binding. Future examination of this hypothesis requires the development of new methods that measure VX‐809 binding directly rather than indirectly, that is, through activity assays.

Taken together, these studies suggest that the zCftr will be useful in modeling the molecular basis for mutation‐related defects and therapeutic interventions for CF. Strategic mutagenesis studies of the F507del‐zCftr, identifying those residues that are critical in conferring lumacaftor activity will be invaluable for understanding the mechanism of action of this compound. Furthermore, small molecule screens for correctors of the F507del‐zCftr processing defect will be useful for identifying chemicals that have the potential to work differently than lumacaftor and may complement its activity. Given the profound effect of temperature in rescuing the processing and functional defects of F507del‐zCftr, this organism may also prove extremely useful in defining critical proteostatic mechanisms that govern the proper assembly of the major mutant in vivo. Indeed, in vivo screens using zebrafish are becoming more commonplace^66^, ^67^ and given the description of a CF‐relevant phenotype in the Cftr KO zebrafish by Bagnat and colleagues,^17^ a phenotypic screen of chaperone protein targets of potential benefit as therapeutic targets for CFTR folding mutants could be envisioned.

## CONFLICT OF INTEREST

None of the authors have a conflict of interest to declare.

## AUTHOR CONTRIBUTIONS

O. Laselva, Z. Ivakine, and CE Bear designed research; O. Laselva, S. Erwood, and Kai Du. performed research and analyzed data; O. Laselva and CE Bear wrote the paper.

## References

[1] Boat TF , Cheng PW . Epithelial cell dysfunction in cystic fibrosis: implications for airways disease. Acta Paediatr Scand Suppl. 1989;363, 25‐29. discussion 25–30. 2701921 10.1111/apa.1989.78.s363.25

[2] Rommens J , Iannuzzi M , Kerem B , et al. Identification of the cystic fibrosis gene: chromosome walking and jumping. Science. 1989;245:1059‐1065. 2772657 10.1126/science.2772657

[3] Anderson MP , Gregory RJ , Thompson S , et al. Demonstration that CFTR is a chloride channel by alteration of its anion selectivity. Science. 1991;253:202‐205. 1712984 10.1126/science.1712984

[4] Huang P , Gilmore E , Kultgen P , Barnes P , Milgram S , Stutts MJ . Local regulation of cystic fibrosis transmembrane regulator and epithelial sodium channel in airway epithelium. Proc Am Thorac Soc. 2004;1:33‐37. 16113409 10.1513/pats.2306012

[5] Li C , Naren AP . Macromolecular complexes of cystic fibrosis transmembrane conductance regulator and its interacting partners. Pharmacol Ther. 2005;108:208‐223. 15936089 10.1016/j.pharmthera.2005.04.004

[6] Boucher RC . Cystic fibrosis: a disease of vulnerability to airway surface dehydration. Trends Mol Med. 2007;13:231‐240. 17524805 10.1016/j.molmed.2007.05.001

[7] Stoltz DA , Meyerholz DK , Welsh MJ . Origins of cystic fibrosis lung disease. N Engl J Med. 2015;372:351‐362. 25607428 10.1056/NEJMra1300109 PMC4916857

[8] Riordan JR , Rommens J , Kerem B , et al. Identification of the cystic fibrosis gene: cloning and characterization of complementary DNA. Science. 1989;245:1066‐1073. 2475911 10.1126/science.2475911

[9] Dawson RJ , Locher KP . Structure of a bacterial multidrug ABC transporter. Nature. 2006;443:180‐185. 16943773 10.1038/nature05155

[10] Dawson RJ , Locher KP . Structure of the multidrug ABC transporter Sav 1866 from Staphylococcus aureus in complex with AMP‐PNP. FEBS Lett. 2007;581:935‐938. 17303126 10.1016/j.febslet.2007.01.073

[11] Mornon JP , Lehn P , Callebaut I . Atomic model of human cystic fibrosis transmembrane conductance regulator: membrane‐spanning domains and coupling interfaces. Cell Mol Life Sci. 2008;65:2594‐2612. 18597042 10.1007/s00018-008-8249-1 PMC11131860

[12] Zhang Z , Chen J . Atomic structure of the cystic fibrosis transmembrane conductance regulator. Cell. 2016;167(1586–1597):e1589. 10.1016/j.cell.2016.11.014 27912062

[13] Zhang Z , Liu F , Chen J . Conformational changes of CFTR upon phosphorylation and ATP binding. Cell. 2017;170(483–491):e488. 10.1016/j.cell.2017.06.041 28735752

[14] Liu F , Zhang Z , Csanady L , Gadsby DC , Chen J . Molecular structure of the human CFTR ion channel. Cell. 2017;169(85–95):e88. 10.1016/j.cell.2017.02.024 28340353

[15] Zhang Z , Liu F , Chen J . Molecular structure of the ATP‐bound, phosphorylated human CFTR. Proc Natl Acad Sci U S A. 2018;115:12757‐12762. 30459277 10.1073/pnas.1815287115 PMC6294961

[16] Zhang J , Yu YC , Yeh JT , Hwang TC . Functional characterization reveals that zebrafish CFTR prefers to occupy closed channel conformations. PLoS ONE. 2018;13:e0209862. 30596737 10.1371/journal.pone.0209862 PMC6312236

[17] Navis A , Marjoram L , Bagnat M . Cftr controls lumen expansion and function of Kupffer's vesicle in zebrafish. Development. 2013;140:1703‐1712. 23487313 10.1242/dev.091819 PMC3621488

[18] Navis A , Bagnat M . Loss of cftr function leads to pancreatic destruction in larval zebrafish. Dev Biol. 2015;399:237‐248. 25592226 10.1016/j.ydbio.2014.12.034 PMC4765326

[19] Du K , Sharma M , Lukacs GL . The DeltaF508 cystic fibrosis mutation impairs domain‐domain interactions and arrests post‐translational folding of CFTR. Nat Struct Mol Biol. 2005;12:17‐25. 15619635 10.1038/nsmb882

[20] Thibodeau PH , Brautigam CA , Machius M , Thomas PJ . Side chain and backbone contributions of Phe508 to CFTR folding. Nat Struct Mol Biol. 2005;12:10‐16. 15619636 10.1038/nsmb881 PMC3516198

[21] Wang C , Protasevich I , Yang Z , et al. Integrated biophysical studies implicate partial unfolding of NBD1 of CFTR in the molecular pathogenesis of F508del cystic fibrosis. Protein Sci. 2010;19:1932‐1947. 20687163 10.1002/pro.480 PMC2998727

[22] Protasevich I , Yang Z , Wang C , et al. Thermal unfolding studies show the disease causing F508del mutation in CFTR thermodynamically destabilizes nucleotide‐binding domain 1. Protein Sci. 2010;19:1917‐1931. 20687133 10.1002/pro.479 PMC2998726

[23] Lukacs GL , Verkman AS . CFTR: folding, misfolding and correcting the DeltaF508 conformational defect. Trends Mol Med. 2012;18:81‐91. 22138491 10.1016/j.molmed.2011.10.003 PMC3643519

[24] Ward CL , Omura S , Kopito RR . Degradation of CFTR by the ubiquitin‐proteasome pathway. Cell. 1995;83:121‐127. 7553863 10.1016/0092-8674(95)90240-6

[25] Jensen TJ , Loo MA , Pind S , Williams DB , Goldberg AL , Riordan JR . Multiple proteolytic systems, including the proteasome, contribute to CFTR processing. Cell. 1995;83:129‐135. 7553864 10.1016/0092-8674(95)90241-4

[26] Cholon DM , O'Neal WK , Randell SH , Riordan JR , Gentzsch M . Modulation of endocytic trafficking and apical stability of CFTR in primary human airway epithelial cultures. Am J Physiol Lung Cell Mol Physiol. 2010;298:L304‐314. 20008117 10.1152/ajplung.00016.2009 PMC2838667

[27] Denning GM , Anderson MP , Amara JF , Marshall J , Smith AE , Welsh MJ . Processing of mutant cystic fibrosis transmembrane conductance regulator is temperature‐sensitive. Nature. 1992;358:761‐764. 1380673 10.1038/358761a0

[28] Sharma M , Benharouga M , Hu W , Lukacs GL . Conformational and temperature‐sensitive stability defects of the delta F508 cystic fibrosis transmembrane conductance regulator in post‐endoplasmic reticulum compartments. J Biol Chem. 2001;276:8942‐8950. 11124952 10.1074/jbc.M009172200

[29] Van Goor F , Hadida S , Grootenhuis PD , et al. Rescue of CF airway epithelial cell function in vitro by a CFTR potentiator, VX‐770. Proc Natl Acad Sci USA. 2009;106:18825‐18830. 19846789 10.1073/pnas.0904709106 PMC2773991

[30] Van Goor F , Hadida S , Grootenhuis PD , et al. Correction of the F508del‐CFTR protein processing defect in vitro by the investigational drug VX‐809. Proc Natl Acad Sci USA. 2011;108:18843‐18848. 21976485 10.1073/pnas.1105787108 PMC3219147

[31] Wainwright CE , Elborn JS , Ramsey BW , et al. Lumacaftor‐ivacaftor in patients with cystic fibrosis homozygous for Phe508del CFTR. N Engl J Med. 2015;373:220‐231. 26510034 10.1056/NEJMc1510466

[32] Eckford PD , Li C , Ramjeesingh M , Bear CE . Cystic fibrosis transmembrane conductance regulator (CFTR) potentiator VX‐770 (ivacaftor) opens the defective channel gate of mutant CFTR in a phosphorylation‐dependent but ATP‐independent manner. J Biol Chem. 2012;287:36639‐36649. 22942289 10.1074/jbc.M112.393637 PMC3481266

[33] Eckford PD , Ramjeesingh M , Molinski S , et al. VX‐809 and related corrector compounds exhibit secondary activity stabilizing active F508del‐CFTR after its partial rescue to the cell surface. Chem Biol. 2014;21:666‐678. 24726831 10.1016/j.chembiol.2014.02.021

[34] Farinha CM , Sousa M , Canato S , Schmidt A , Uliyakina I , Amaral MD . Increased efficacy of VX‐809 in different cellular systems results from an early stabilization effect of F508del‐CFTR. Pharmacol Res Perspect. 2015;3:e00152. 26171232 10.1002/prp2.152 PMC4492728

[35] Ren HY , Grove DE , De La Rosa O , et al. VX‐809 corrects folding defects in cystic fibrosis transmembrane conductance regulator protein through action on membrane‐spanning domain 1. Mol Biol Cell. 2013;24:3016‐3024. 23924900 10.1091/mbc.E13-05-0240 PMC3784376

[36] Loo TW , Bartlett MC , Clarke DM . Corrector VX‐809 stabilizes the first transmembrane domain of CFTR. Biochem Pharmacol. 2013;86:612‐619. 23835419 10.1016/j.bcp.2013.06.028

[37] Abbattiscianni AC , Favia M , Mancini MT , et al. Correctors of mutant CFTR enhance subcortical cAMP‐PKA signaling through modulating ezrin phosphorylation and cytoskeleton organization. J Cell Sci. 2016;129:1128‐1140. 26823603 10.1242/jcs.177907

[38] Jih KY , Hwang TC . Vx‐770 potentiates CFTR function by promoting decoupling between the gating cycle and ATP hydrolysis cycle. Proc Natl Acad Sci USA. 2013;110:4404‐4409. 23440202 10.1073/pnas.1215982110 PMC3600496

[39] Meng X , Wang Y , Wang X , et al. Two small molecules restore stability to a subpopulation of the cystic fibrosis transmembrane conductance regulator with the predominant disease‐causing mutation. J Biol Chem. 2017;292:3706‐3719. 28087700 10.1074/jbc.M116.751537 PMC5339754

[40] Laselva O , Molinski S , Casavola V , Bear CE . Correctors of the major cystic fibrosis mutant interact through membrane‐spanning domains. Mol Pharmacol. 2018;93:612‐618. 29618585 10.1124/mol.118.111799

[41] Liu F , Zhang Z , Levit A , et al. Structural identification of a hotspot on CFTR for potentiation. Science. 2019;364:1184‐1188. 31221859 10.1126/science.aaw7611 PMC7184887

[42] D'Antonio C , Molinski S , Ahmadi S , Huan LJ , Wellhauser L , Bear CE . Conformational defects underlie proteasomal degradation of Dent's disease‐causing mutants of ClC‐5. Biochem J. 2013;452:391‐400. 23566014 10.1042/BJ20121848

[43] Molinski SV , Ahmadi S , Hung M , Bear CE . Facilitating structure‐function studies of CFTR modulator sites with efficiencies in mutagenesis and functional screening. J Biomol Screen. 2015;20:1204‐1217. 26385858 10.1177/1087057115605834

[44] Laselva O , Molinski S , Casavola V , Bear CE . The investigational cystic fibrosis drug trimethylangelicin directly modulates CFTR by stabilizing the first membrane‐spanning domain. Biochem Pharmacol. 2016;119:85‐92. 27614011 10.1016/j.bcp.2016.09.005

[45] Molinski SV , Ahmadi S , Ip W , et al. Orkambi(R) and amplifier co‐therapy improves function from a rare CFTR mutation in gene‐edited cells and patient tissue. EMBO Mol Med. 2017;9:1224‐1243. 28667089 10.15252/emmm.201607137 PMC5582412

[46] Molinski SV , Shahani VM , Subramanian AS , et al. Comprehensive mapping of cystic fibrosis mutations to CFTR protein identifies mutation clusters and molecular docking predicts corrector binding site. Proteins. 2018;86:833‐843. 29569753 10.1002/prot.25496

[47] Chin S , Ramjeesingh M , Hung M , et al. Cholesterol interaction directly enhances intrinsic activity of the cystic fibrosis transmembrane conductance regulator (CFTR). Cells. 2019;8:8. 10.3390/cells8080804 PMC6721619 31370288

[48] Ahmadi S , Bozoky Z , Di Paola M , et al. Phenotypic profiling of CFTR modulators in patient‐derived respiratory epithelia. NPJ Genom Med. 2017;2:12. 28649446 10.1038/s41525-017-0015-6 PMC5481189

[49] Laselva O , Marzaro G , Vaccarin C , et al. Molecular mechanism of action of trimethylangelicin derivatives as CFTR modulators. Front Pharmacol. 2018;9:719. 30022950 10.3389/fphar.2018.00719 PMC6039571

[50] Cheng SH , Gregory RJ , Marshall J , et al. Defective intracellular transport and processing of CFTR is the molecular basis of most cystic fibrosis. Cell. 1990;63:827‐834. 1699669 10.1016/0092-8674(90)90148-8

[51] Heda GD , Tanwani M , Marino CR . The Delta F508 mutation shortens the biochemical half‐life of plasma membrane CFTR in polarized epithelial cells. Am J Physiol Cell Physiol. 2001;280:C166‐174. 11121388 10.1152/ajpcell.2001.280.1.C166

[52] Zhang Q , Kopp M , Babiak I , Fernandes J . Low incubation temperature during early development negatively affects survival and related innate immune processes in zebrafish larvae exposed to lipopolysaccharide. Sci Rep. 2018;8:4142. 29515182 10.1038/s41598-018-22288-8 PMC5841277

[53] Aleksandrov AA , Kota P , Cui L , et al. Allosteric modulation balances thermodynamic stability and restores function of DeltaF508 CFTR. J Mol Biol. 2012;419:41‐60. 22406676 10.1016/j.jmb.2012.03.001 PMC3891843

[54] Ostedgaard LS , Rogers CS , Dong Q , et al. Processing and function of CFTR‐DeltaF508 are species‐dependent. Proc Natl Acad Sci USA. 2007;104:15370‐15375. 17873061 10.1073/pnas.0706974104 PMC1976592

[55] Cui G , Khazanov N , Stauffer BB , et al. Potentiators exert distinct effects on human, murine, and Xenopus CFTR. Am J Physiol Lung Cell Mol Physiol. 2016;311:L192‐207. 27288484 10.1152/ajplung.00056.2016 PMC5142458

[56] Cai Z , Palmai‐Pallag T , Khuituan P , et al. Impact of the F508del mutation on ovine CFTR, a Cl‐channel with enhanced conductance and ATP‐dependent gating. J Physiol. 2015;593:2427‐2446. 25763566 10.1113/JP270227 PMC4461407

[57] Xavier BM , Hildebrandt E , Jiang F , Ding H , Kappes JC , Urbatsch IL . Substitution of Yor1p NBD1 residues improves the thermal stability of human cystic fibrosis transmembrane conductance regulator. Protein Eng Des Sel. 2017;30:729‐741. 29053845 10.1093/protein/gzx054 PMC5914393

[58] Dong Q , Ostedgaard LS , Rogers C , Vermeer DW , Zhang Y , Welsh MJ . Human‐mouse cystic fibrosis transmembrane conductance regulator (CFTR) chimeras identify regions that partially rescue CFTR‐DeltaF508 processing and alter its gating defect. Proc Natl Acad Sci USA. 2012;109:917‐922. 22210114 10.1073/pnas.1120065109 PMC3271874

[59] Farinha CM , King‐Underwood J , Sousa M , et al. Revertants, low temperature, and correctors reveal the mechanism of F508del‐CFTR rescue by VX‐809 and suggest multiple agents for full correction. Chem Biol. 2013;20:943‐955. 23890012 10.1016/j.chembiol.2013.06.004

[60] DeCarvalho AC , Gansheroff LJ , Teem JL . Mutations in the nucleotide binding domain 1 signature motif region rescue processing and functional defects of cystic fibrosis transmembrane conductance regulator delta f508. J Biol Chem. 2002;277:35896‐35905. 12110684 10.1074/jbc.M205644200

[61] Hoelen H , Kleizen B , Schmidt A , et al. The primary folding defect and rescue of DeltaF508 CFTR emerge during translation of the mutant domain. PLoS ONE. 2010;5:e15458. 21152102 10.1371/journal.pone.0015458 PMC2994901

[62] Bose SJ , Bijvelds M , Wang Y , et al. Differential thermostability and response to cystic fibrosis transmembrane conductance regulator potentiators of human and mouse F508del‐CFTR. Am J Physiol Lung Cell Mol Physiol. 2019;317:L71‐L86. 30969810 10.1152/ajplung.00034.2019 PMC6689747

[63] Okiyoneda T , Veit G , Dekkers JF , et al. Mechanism‐based corrector combination restores DeltaF508‐CFTR folding and function. Nat Chem Biol. 2013;9:444‐454. 23666117 10.1038/nchembio.1253 PMC3840170

[64] Hudson RP , Dawson JE , Chong PA , et al. Direct binding of the corrector VX‐809 to Human CFTR NBD1: evidence of an allosteric coupling between the binding site and the NBD1:CL4 interface. Mol Pharmacol. 2017;92:124‐135. 28546419 10.1124/mol.117.108373

[65] He L , Kota P , Aleksandrov AA , et al. Correctors of DeltaF508 CFTR restore global conformational maturation without thermally stabilizing the mutant protein. FASEB J. 2013;27:536‐545. 23104983 10.1096/fj.12-216119 PMC3545534

[66] Early JJ , Cole KL , Williamson JM , et al. An automated high‐resolution in vivo screen in zebrafish to identify chemical regulators of myelination. Elife 2018;7. 10.7554/eLife.35136 PMC6056238 29979149

[67] Cafora M , Deflorian G , Forti F , et al. Phage therapy against Pseudomonas aeruginosa infections in a cystic fibrosis zebrafish model. Sci Rep. 2019;9:1527. 30728389 10.1038/s41598-018-37636-x PMC6365511

