# Supplementary figures and images for: Activity of lumacaftor is not conserved in zebrafish Cftr bearing the major cystic fibrosis‐causing mutation
Source: FASEB Bioadv. 2019 Sep 18;1(10):661–70. doi: 10.1096/fba.2019-00039 (PMC6996396; doi:10.1096/fba.2019-00039)

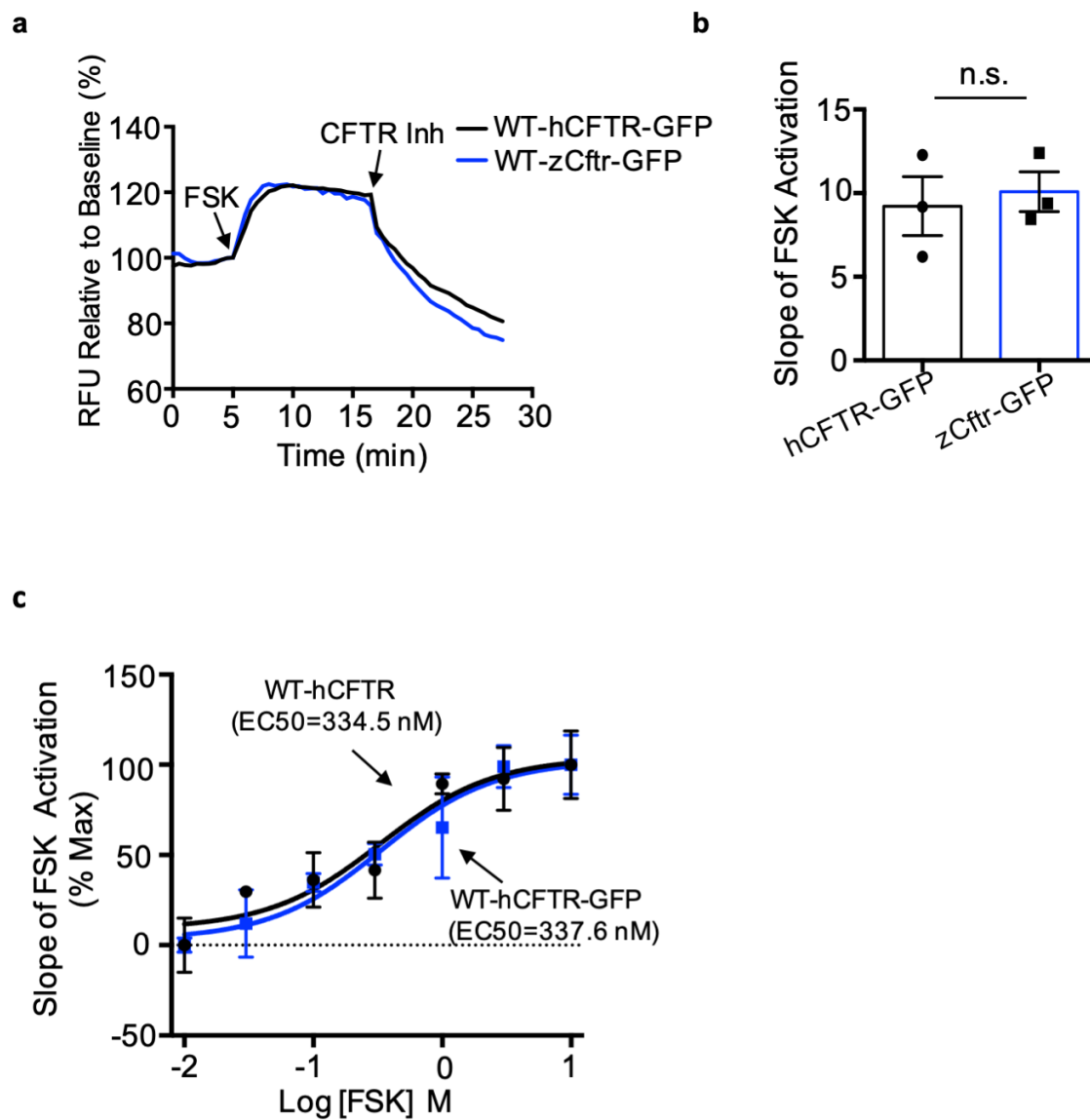

FIGURE S1

Supplement: Supplementary file 1 [file FBA2-1-661-s001.pdf]

**a**

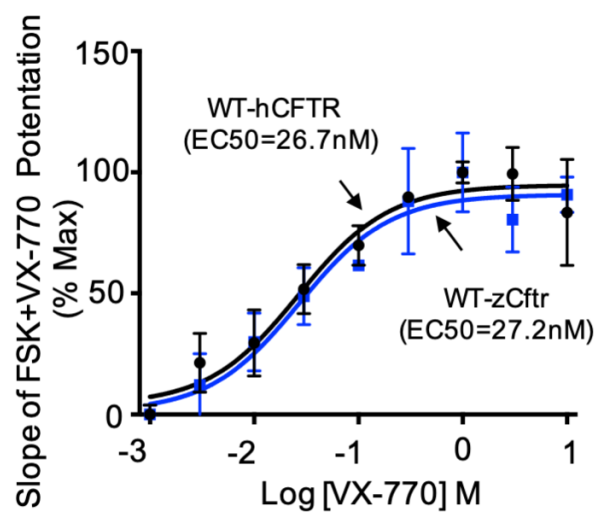

**FIGURE S2**

Supplement: Supplementary file 2 [file FBA2-1-661-s002.pdf]

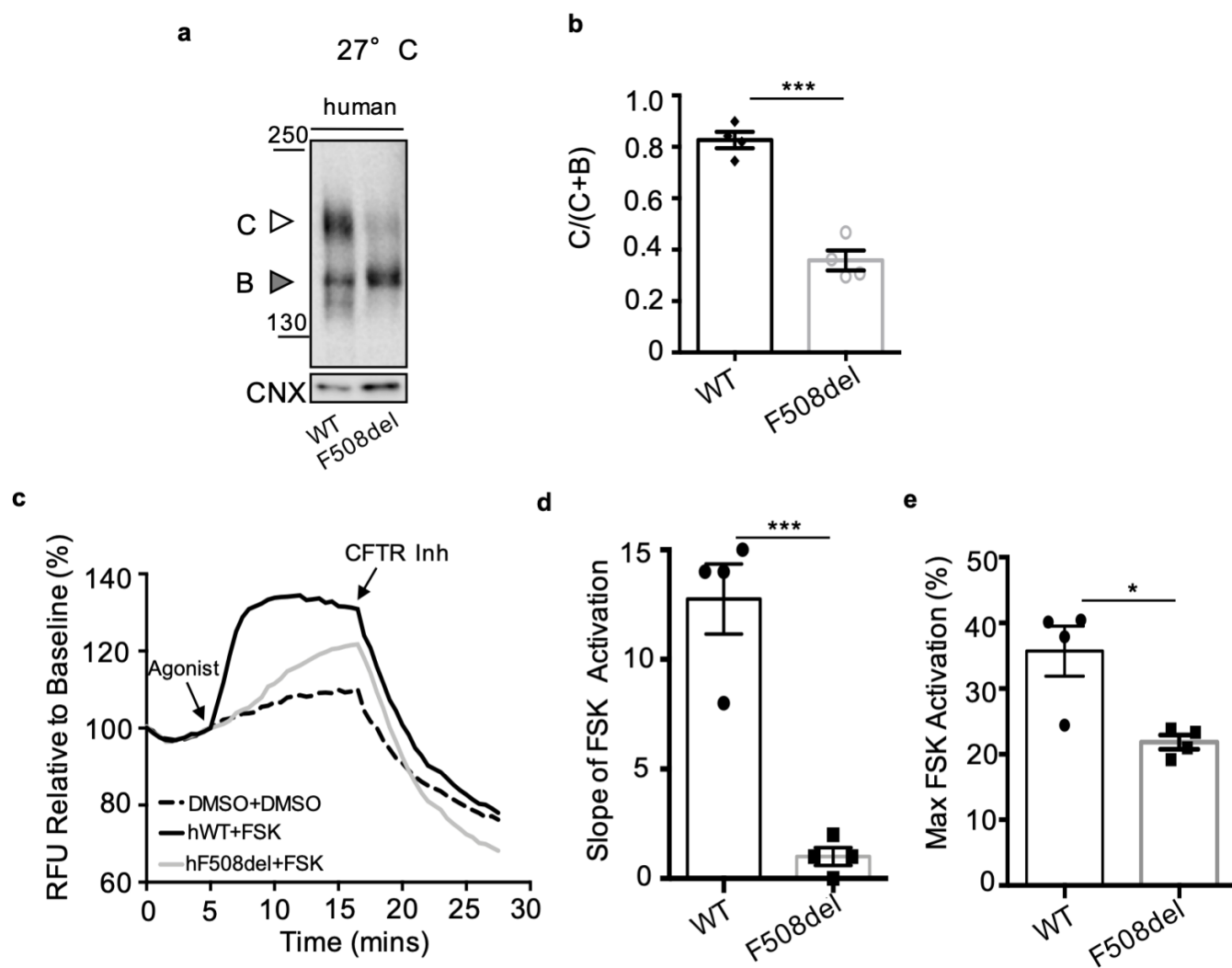

FIGURE S3

Supplement: Supplementary file 3 [file FBA2-1-661-s003.pdf]
