# Supplementary material for: Activity of lumacaftor is not conserved in zebrafish Cftr bearing the major cystic fibrosis‐causing mutation
Source: FASEB Bioadv. 2019 Sep 18;1(10):661–70. doi: 10.1096/fba.2019-00039 (PMC6996396; doi:10.1096/fba.2019-00039)
Supplement: Supplementary file 4 [file FBA2-1-661-s004.pdf]

a

```
zCFTR  479  GKIRHSGRISYSSQTAWIMPGTIRDNILFGLTYDEYRYKSVVKACQLEEDLAALPEKDK  T 538
hCFTR  480  GKIKHSGRISFCSQFSWIMPGTIKENII FGVSYDEYRYRSV IKACQLEEDISKFAEKDN  I 539
```

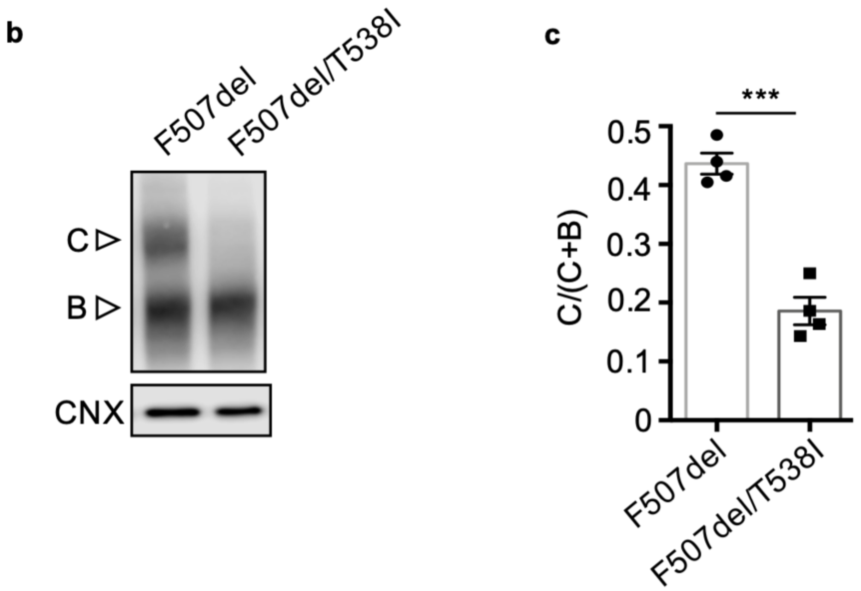

FIGURE S4
